# Advancing cooperation in Health Technology Assessment in Europe: insights from the EUnetHTA 21 project amidst the evolving legal landscape of European HTA

**DOI:** 10.1017/S0266462324004689

**Published:** 2024-12-12

**Authors:** Irene Urbina, Roisin Adams, Judith Fernandez, Anne Willemsen, Niklas Hedberg, Alric Rüther

**Affiliations:** 1 Institute for Quality and Efficiency in Healthcare, Cologne, Germany; 2 National Centre for Pharmacoeconomics, Dublin, Ireland; 3 Haute Autorité de Santé, La Plaine Saint-Denis, France; 4 Zorginstituut Nederland, Diemen, The Netherlands; 5 Tandvårds- och Läkemedelsförmånsverket, Stockholm, Sweden

**Keywords:** health technology assessment, Europe, European Regulation, international networks, EUnetHTA, health policy

## Abstract

Health Technology Assessment (HTA) in Europe has undergone significant evolution, culminating in the adoption of Regulation (EU) 2021/2282 on HTA (HTAR) aimed at fostering sustainable collaboration in HTA at the European Union (EU) level. The EUnetHTA 21 project, a 2-year initiative, was commissioned to address key methodological issues and prepare for the implementation of the HTAR. This commentary documents the outcomes of the EUnetHTA 21 project, focusing on Joint Clinical Assessments (JCAs), while analyzing challenges encountered and lessons learned for future collaboration under the HTAR. The EUnetHTA 21 consortium, comprising thirteen European HTA bodies, developed twenty guidance documents and thirteen templates, refining methods and procedures for joint work in HTA at EU level. Pilot JCAs and Joint Scientific Consultations were conducted to test these materials. Lessons learned from this experience emphasize the importance of inclusive consensus building, effective time and resource management, capacity building, and continuous quality improvement. The project’s realization underscores a collective commitment among HTA bodies to continue to collaborate, now under a legal framework. Recommendations from the project, along with experiences gained from previous European Network for HTA (EUnetHTA) Joint Actions, provide a foundation for developing guidance for EU-HTA under the HTAR. Further proactive efforts at national and central levels are essential to coordinate and ensure a sustainable cooperation. The EUnetHTA 21 experience provides valuable insights for advancing cooperation in HTA under the HTAR, aiming to improve the quality of HTA, avoid duplication, and ultimately enhance patient access to safe and effective health technologies in the EU.

## Background

Over the course of more than two decades, Health Technology Assessment (HTA) bodies across Europe have engaged in voluntary project-based cooperation cofinanced by the European Union (EU), including the formation of the European Network for HTA (EUnetHTA). The aim of these endeavors gradually evolved toward establishing a permanent and sustainable framework for collaboration in HTA in Europe, ultimately contributing to the formulation of Regulation (EU) 2021/2282 on HTA (HTAR), adopted in December 2021 (1).

The HTAR aims to establish a sustainable legal and financial framework for European-level cooperation in HTA (EU-HTA), with a phased implementation starting from January 2025. The joint work specified in the HTAR includes Joint Clinical Assessments (JCAs) of specific health technologies, as well as Joint Scientific Consultations (JSCs), and identification of emerging health technologies. Beyond this, the regulation allows Member States of the EU and the European Economic Area (hereafter “Member States”) to undertake voluntary cooperation on HTA (2).

Collaboration within the HTAR framework is centrally steered by Member States through the Coordination Group on HTA (HTACG). The group, formally established in June 2022, plays a pivotal role in providing strategic direction and overseeing subgroups dedicated to work in JCA, JSC, identification of emerging health technologies, and development of methodological and procedural guidance. The HTACG is ultimately responsible for the adoption of methodological guidance and procedural steps for the conduct of joint work as well as for the endorsement of outputs of the collaboration under the HTAR. Moreover, the HTAR specifies that responsibility for the secretariat function for the HTACG and its subgroups lies with the European Commission. This role includes, among other tasks, providing administrative, technical, and information technology (IT) support for the execution of EU-HTA activities under the regulation.

In anticipation of the HTAR adoption and with the intention of supporting the preparations for its implementation, in early 2021, the EU tendered a 2-year service contract that was awarded by the European Health and Executive Agency (HaDEA) to EUnetHTA 21, a consortium comprising thirteen European HTA bodies with prior involvement in EUnetHTA. The subject of the call for tenders was “to address some key methodological issues which have been identified as instrumental to foster the value of EU cooperation on HTA and provide input to a potential new legal framework on HTA” (3).

The purpose of this commentary is to document the EUnetHTA 21 project, highlighting its outcomes, in particular those related to JCA, while critically analyzing encountered challenges and distilling key lessons learned for further collaboration under the HTAR. Additionally, it seeks to showcase the project’s significance within the broader European HTA landscape.

## EUnetHTA 21 project overview

The EUnetHTA 21 project took place over the course of 24 months, initiating in September 2021. The consortium responsible for its development consisted of thirteen European HTA bodies, each with prior participation in EUnetHTA Joint Actions: Agencia Española de Medicamentos y Productos Sanitarios (AEMPS, Spain), Austrian Institute for HTA (AIHTA, Austria), Belgian Health Care Knowledge Centre (KCE, Belgium), Federal Joint Committee (G-BA, Germany), Haute Autorité de Santé (HAS, France), Institute for Quality and Efficiency in Health Care (IQWiG, Germany), Italian Medicines Agency (AIFA, Italy), National Authority of Medicines and Health Products I.P. (INFARMED, Portugal), National Centre for Pharmacoeconomics, St. James Hospital (NCPE, Ireland), National Institute of Pharmacy and Nutrition (NIPN, Hungary), Norwegian Medicines Agency (NOMA, Norway), Dental and Pharmaceutical Benefits Agency (TLV, Sweden), and Zorginstituut Nederland (ZIN, The Netherlands), with ZIN serving as the consortium coordinator.

Building on the achievements and lessons learned from the three EUnetHTA Joint Actions (4) and considering the developments of the legal framework at the time and, later, the final text of the HTAR, the EUnetHTA 21 project had two main goals: refining methods and procedures for joint work and testing them in pilot JCAs and JSCs.

**Figure 1.**
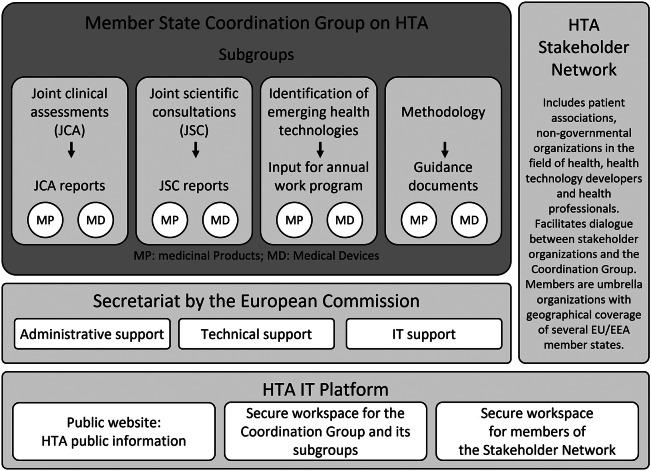
Governance structure for the European Health Technology Assessment Regulation (28).

**Figure 2.**
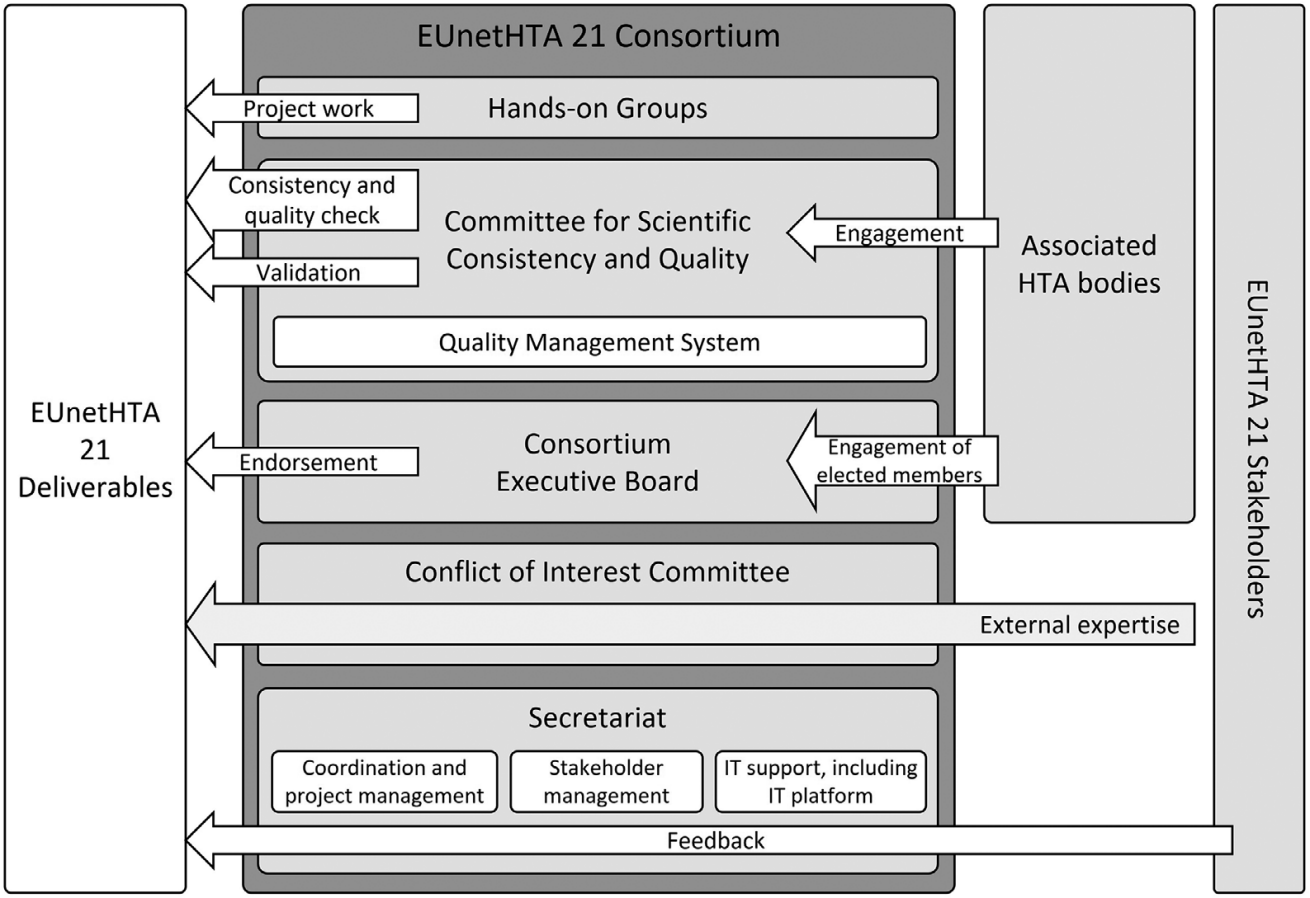
Governance structure and actor involvement in the EUnetHTA 21 project.

To mirror the anticipated structure of EU-HTA governance (see Figure 1), the project adopted a similar setup (see Figure 2). It featured a strategic group, referred to as the “consortium executive board” (CEB), and a technical counterpart known as the “committee for scientific consistency and quality” (CSCQ), drawing parallels with the HTACG and its subgroups. By practically implementing this structure for its operations, the consortium gained valuable insights for future governance considerations. Furthermore, all functions under the project were coordinated and supported by a central secretariat. A shared IT platform, reinforced with multiple layers of data security, facilitated collaborative work.

Acknowledging the limitations inherent in being a relatively small group of organizations, EUnetHTA 21 actively engaged nonconsortium European HTA bodies, referred to as “associated HTA bodies.” These bodies reviewed project outputs and actively contributed to technical discussions on a voluntary basis as part of the CSCQ. A specific subset of these organizations, so-called “elected members,” took part in the strategic discussions of the project executive board, albeit without voting privileges. Additionally, the consortium anticipated public consultations on all publicly available deliverables, in which feedback was obtained from stakeholder organizations, including patient associations, healthcare professional associations, health technology developers, academia, and extra-European HTA bodies. This inclusive approach aimed to incorporate a diverse range of perspectives, ensuring a comprehensive shaping of the project’s outcomes.

## EUnetHTA 21 project outcomes

The EUnetHTA 21 consortium produced twenty guidance documents including methodological, practical, and procedural guidelines, along with thirteen templates for joint work in HTA in Europe (see Table 1).

**Table 1.** Results of the EUnetHTA 21 project (29)

Scope	Guidance documents	Templates	Others
JCA	JCA timelines (6) Synthesis of national requirements and framework for the assessment of high-risk medical devices and in vitro diagnostics (30) Guidance for European database on medical devices (EUDAMED)-based topic identification selection and prioritisation (TISP) process (31) Scoping process (21) Outcomes (endpoints) (32) Submission Dossier Guidance (12) Assessment Report Guidance (16) Direct and indirect comparisons (methodological guideline) (33) Direct and indirect comparisons (practical guideline) (34) Applicability of evidence (35) Validity of clinical studies (36) Procedure and framework for the factual accuracy check (37)	Input from HCP – external expert (38) Input from HCP – stakeholder (39) Input from patient – external expert (40) Input from patient – stakeholder (41) EUDAMED data reporting template (31) Submission Dossier Template (13–15) Assessment Report Template (17)	Production of 2 JCAs – medical devices (7;8) Conduction of 3 “JCAs without health technology developer submission” (JCA scoping/PICO exercises) – medicinal products (9–11)
Transversal deliverables	Guidelines for appointing assessors and co-assessors (42) Resourcing and maintaining HTA-bodies technical expert WGs (43) Handling DOI and ECA forms (44) Guidance on patient and HCP involvement (45) Practical guideline for interaction between health technology developer and HTA bodies (46) Guidance for handling commercially confidential data (47) Procedure for scientific consistency and quality control of EUnetHTA 21 outputs	DOI template ECA template	Interaction with regulators – medicinal products Interaction with regulators – medical devices
JSC	Guidance on parallel EMA/EUnetHTA 21 JSC (48)	Application form template (49) Briefing document template (50) Short study information template (51) EUnetHTA 21 final written recommendations template	Production of 7 parallel EMA/EUnetHTA 21 JSCs

DOI, Declaration of Interest; ECA, EUnetHTA 21 Confidentiality Agreement; EMA, European Medicines Agency; HTA, Health Technology Assessment; HCP, Healthcare Professional; JCA, Joint Clinical Assessment; JSC, Joint Scientific Consultation; WG, working group.

These methodological and procedural deliverables of EUnetHTA 21 were built upon materials from previous stages of the collaboration, that is, Joint Actions, which were systematically organized in a comprehensive Quality Management System (5). In contrast to these previous outputs intended for use under a voluntary framework, the EUnetHTA 21 deliverables constitute recommendations by the consortium for conducting JCAs and JSCs within the context of EU-HTA under the HTAR.

In response to substantial discussions concerning the practical feasibility of the process as outlined by the HTAR, the consortium made the strategic decision to formulate suggested timelines for the conducting of JCAs of medicinal products (6), supplementing the initially planned deliverables.

The consortium tested the newly developed guidance and templates in two pilot JCAs on medical devices (7;8) and seven pilot JSCs on medicinal products. Regrettably, due to the lack of evidence submissions from health technology developers, conducting full pilot JCAs on medicinal products proved unfeasible. Nevertheless, the consortium conducted three pilot JCAs without health technology developer submission (9–11), that is, exercises for testing the EUnetHTA 21 guidance for the JCA scoping process. For this purpose, the consortium selected three medicinal products that had recently received a positive opinion from the Committee for Medicinal Products for Human Use (CHMP) of the European Medicines Agency (EMA). In an effort to align with the initial categories targeted by the HTAR, medicinal products either containing an active substance indicated for the treatment of cancer, regulated as advanced therapy medicinal product, or designated as orphan medicinal product were selected.

Additionally, the EUnetHTA 21 consortium actively engaged with regulatory actors delineated in the HTAR, including the EMA, which also serves as the Secretariat of the Expert Panels, the Medical Devices Coordination Group, and notified bodies, fostering mutual understanding, discussing potential areas for cooperation and exploring ways for establishing such cooperation under the HTAR.

Operating within a well-defined scope, the EUnetHTA 21 project has brought forth several questions of diverse nature requiring further exploration. Key considerations include topics such as the potential establishment of specific working groups (WGs) or committees as deemed necessary, further guidance explaining which data could be used to answer a PICO (population – intervention – comparator – outcome) question, formal agreements for cooperation with regulators, criteria for the allocation of joint work projects among willing and suitable HTA bodies, formulation of strategies to handle incomplete JCA dossiers, setting specifications for joint work on medical devices, and devising approaches to address potential deviations from JCA timelines, among other matters requiring thorough examination.

## Considerations for policy and practice

The development of EUnetHTA 21 deliverables was guided by several key principles outlined in the HTAR, which facilitate the use of JCA reports by Member States as input for the critical appraisal of evidence that can be integrated into decision making processes at national and/or local level in areas such as pricing, reimbursement, budgeting, priority setting, benefit package design, and so forth. Firstly, it is mandated to give due consideration to JCA reports for any HTA activities at Member State level, as stipulated in Article 13(1). Additionally, Article 2 of the regulation underscores that each Member State has the “competence to draw conclusions on the relative effectiveness of health technologies or to take decisions on the use of a health technology in their specific national health context.” Furthermore, JCA reports are required, per Article 9(1) of the HTAR, to abstain from containing value judgments or conclusions on the overall clinical added value of the assessed health technology; instead focusing solely on a scientific analysis of relative effects of the health technology and the certainty of these effects, considering the strengths and limitations of the available evidence. Moreover, these reports must be based on scientific evidence submitted by health technology developers and adhere to inclusivity, that is, the scope of each report shall align with the information needs of all Member States, as determined respectively by Article 9 and Article 8(6) of the regulation (2).

Article 4(4) of the HTAR stipulates that methodologies and procedures already developed by the EUnetHTA Joint Actions undergo formal consideration by the HTACG and its subgroups. These are meant to lay the groundwork for developing methodological and procedural guidance for collaborative work under the HTAR. Furthermore, the European Commission indicates in the tender specifications for the EUnetHTA 21 project that materials produced in its framework are destined to provide relevant input to the (at the time of the tender publication “potential new”) legal framework on HTA (3). Ultimately, the HTACG is tasked with the official adoption of methodological guidance and procedural steps for joint work under the HTAR (2).

To further delineate HTA legislation, the HTAR confers authority upon the European Commission to enact implementing acts according to the specifications outlined in Articles 32 to 34. Certain EUnetHTA 21 deliverables were specifically drafted to facilitate their preparation, for instance, the Submission Dossier Guidance (12) and Template (13–15) and the Assessment Report Guidance (16) and Template (17). The preparation and adoption of such acts involve an examination procedure prescribed by Regulation (EU) 182/2011, which requires the European Commission to be supported by a dedicated committee comprising representatives from Member States (2;18), known as the Comitology Committee on HTA (19).

## Challenges and lessons learned: the example of the guidance for the JCA scoping process

The development and testing of guidance for the JCA scoping process serve as an example of collaboration in the EUnetHTA 21 project context. This experience sheds light on encountered challenges and essential lessons learned, offering insights valuable for the ongoing development of procedural and methodological guidance for the implementation of the HTAR (see Box 1).

Box 1. Key lessons for further methodological and procedural work and conduct of joint work.

***Key lessons for further methodological and procedural work and conduct of joint work***

- *Bottom-up consensus-building approach and inclusiveness: Collaborative development of drafts in smaller working groups of experts from a variety of Member States ensures a more efficient and timely production approach. Reviews and iterative polishing of drafts involving experts from more Member States ensure a comprehensive perspective.*
- *Time and resource management: Setting realistic timelines for the development of the products and aiming to optimize resource utilization. Considering the formation of specialized subgroups or task forces to address specific methodological issues effectively.*
- *Capacity building: Providing opportunities for capacity-building to address skill gaps, align standards among participants, ensure a shared understanding of basic concepts, and maintain consistent terminology to avoid confusion.*
- *Continuous quality improvement: Recurrently applying the “Plan-Do-Check-Act cycle” (5), embracing an iterative learning process involving discussions, exchanges, and modifications based on practical experiences and insights.*
- *Effective secretariat support: Ensuring that the secretariat is equipped with the necessary resources and capabilities to support the diverse needs of the consortium and facilitate collaborative activities. Key elements, such as effective project management, stakeholder management, communications, IT, and administrative support, play a pivotal role in maintaining adherence to a work plan within a strict schedule.*

In the context of JCA, the scoping process is pivotal. During this phase, Member States collaboratively identify the parameters for each assessment, that is, the assessment scope. As outlined in the HTAR, the assessment scope must be inclusive, reflecting the information, data, analyses, and other evidence needs of Member States to be submitted by the health technology developer in the dossier serving as the basis for an assessment. Policy questions must be formulated in the assessment scope as research questions requiring specific sets of scientific evidence using the PICO framework (20). The EUnetHTA 21 guidance for the JCA scoping process describes the methods and key steps involved, as well as the data presentation considering the definition of PICO(s). It divides the scoping process into three consecutive steps: PICO survey, consolidation, and validation of the assessment scope (21).

The guidance for the JCA scoping process, like all EUnetHTA 21 methodological and procedural project deliverables, was collaboratively developed through a bottom-up consensus-building approach. A dedicated project team (“hands-on group”) was responsible for the development of each one of these deliverables. Each team comprised individuals with specific skills and expertise relevant to the topic of each deliverable, representing different consortium members. The team structure included an authoring HTA body leading the activity, a co-authoring HTA body providing support, and additional project team members contributing input. Methodological discussions within the team led to the drafting of each document, which subsequently underwent comprehensive review by all consortium members and associated HTA bodies, as well as a public consultation. This inclusive process allowed for iterative refinement of deliverables, aligning them with the diverse needs of HTA procedures across Europe. However, the project faced notable time constraints, with the reviewing process proving burdensome due to the volume of project outputs and, accordingly, the volume of comments on each of the deliverables.

The effective completion of the project was heavily reliant on centralized overarching structures that facilitated joint work, reviews, and public consultations. Adhering to the project’s ambitious schedule posed challenges, and the achievement of each milestone was made possible through robust coordination and secretariat support. The secretariat team, based at the coordinating organization of the consortium, played a crucial role, handling project management, stakeholder engagement, communication, and administrative duties to ensure streamlined operations and documentation processes. Transparent channels of communication between the secretariat and consortium members promoted timely dissemination of information and logistical support. While the shared IT platform established for the project proved instrumental as a primary tool for collaborative work, it became clear that a more sophisticated system would be necessary to support the complex joint work under the HTAR; this need arose from challenges concerning structure, functionalities, and compatibility with reference management software.

The guidance for the JCA scoping process underwent testing through the piloting of the process by the consortium and associated HTA bodies − twice with medical devices and thrice with medicinal products, facilitated by a dedicated assessment team in each case. Although the practical application was significantly time-consuming and presented substantial challenges, including the absence of consensus on fundamental terminology and a lack of experience of some partners in formulating policy questions as research questions requiring specific sets of scientific evidence using the PICO framework, these challenges provided invaluable opportunities for improvement.

The initial inconsistencies in defining research questions underscored the fundamental need for capacity-building opportunities to align standards among participants. This scenario prompted further discussions about information requirements necessary for decision making across Member States, emphasizing the importance of clarity and simplicity in this aspect. Moreover, significant efforts were dedicated to harmonizing the understanding of fundamental concepts pertaining to the definition of research questions within the JCA context. To potentiate capacity building, the assessment teams shared insights with the broader group on the first-hand application of the guidance for scope consolidation, aiding the group’s understanding of the process and improving the formulation of research questions in the PICO survey. These discussions led to modifications of the guidance based on learnings from the scoping process tests. The revised version of the guidance included more detailed guidance for consistent research question formulation using the PICO framework, a hint for a potential PICO consolidation WG (PC-WG), a suggestion to promote direct exchanges between authors of a JCA and the potential PC-WG with the representatives of each Member State responsible for the identification of the national PICO question(s).

Addressing fundamental methodological issues encountered challenges during the guidance’s development and testing stages. The brevity of meetings and the large size of the CSCQ group hindered thorough preparation of solutions. A need emerged to explore methods to streamline discussions within this body, resulting in discussions on selected matters conducted by a smaller WG consisting of CEB and CSCQ chairs and methodological experts, in which sufficient time was dedicated to specific issues. Although resource constraints limited the capacity for this approach, it laid the groundwork for more focused and effective problem-solving.

In conclusion, the piloting of the guidance for the JCA scoping process within the EUnetHTA 21 project framework demonstrated promising outcomes. Through iterative testing and feedback mechanisms, the guidance was refined to better align with the requirements outlined by the HTAR. Each iteration of the pilot projects allowed all participants to gain valuable experience, improving their understanding of the process and the information necessary for decision making, while fostering collaboration. Looking ahead, continued refinement and capacity-building efforts will be essential to ensure the effective implementation of the guidance.

## Concluding remarks and future directions

The EUnetHTA 21 project marks the culmination of a voluntary collaboration that has significantly shaped the landscape of HTA in Europe over more than two decades. These joint efforts have not only facilitated mutual learning and knowledge exchange among HTA bodies, fostering understanding of both the commonalities and distinctions in health care and HTA systems across Member States, but have also advanced common approaches for addressing key aspects of HTA. Moreover, this collaboration has cultivated a climate of trust that served as the foundation for an efficient consensus-building process in the context of the 2-year project, which opportunely delivered documents intended to guide activities under the new legal mandate. This substantial number of deliverables exceeds the output of any previous 2-year frame in a EUnetHTA Joint Action. In this perspective, stressing the importance of a shared foundation for the work of representatives of Member States in the HTACG and its subgroups is imperative, and in this sense, EUnetHTA 21 has effectively facilitated the implementation of the HTAR.

While acknowledging the inherent diversity in healthcare systems, HTA approaches, and decision making frameworks across European countries, the realization of the EUnetHTA 21 project underscores a genuine interest among Member States and HTA bodies in bridging these differences. It reflects a collective commitment to advance collaboratively, strategically avoiding duplication of efforts. There is a clear recognition of the need for factual JCA reports of the highest quality that comprehensively present the available body of clinical evidence on comparative effectiveness and safety, suitable to serve as input for evidence-based decision making processes at the Member State level.

The EUnetHTA 21 experience reaffirms the scientific and technical principles for joint work under a sustainable model of cooperation that were identified from the work under the EUnetHTA Joint Actions and documented by EUnetHTA Joint Action 3 in the White Paper “A Future Model of HTA Cooperation” (4). Furthermore, it provides valuable lessons for the development of the joint groundworks for a mandatory framework with a legal mandate.

Many recommendations generated from learnings from EUnetHTA Joint Actions, particularly those regarding cooperation framework, range of HTA activities, principles of involvement, agency participation, governance, engagement of external actors, and support services, including IT infrastructure (22) can be identified as already incorporated in the framework delineated by the HTAR. Others, such as training and capacity development, need further delineation. These lessons, along with insights from the EUnetHTA 21 experience, should guide the development of the more detailed foundations of EU-HTA, that is, methodological and procedural guidance developed by the HTACG and implementing acts adopted by the European Commission. Certain aspects inherent to the cooperation under the HTAR that were out of scope of the voluntary cooperation within Joint Actions and the service contract will require special consideration and discussion within the HTACG.

In light of these considerations, concerted proactive efforts at the national level are needed to synchronize and adapt national HTA frameworks with the overarching European process, integrating the utilization of JCAs as essential input and ensuring cohesiveness and interoperability. Simultaneously, at the central level, substantial resources are required for effectively managing and coordinating EU-wide cooperation in HTA, thereby facilitating the sustained engagement of all participants.

In considering the broader landscape for medicinal products, transparent sharing of information, including evidence for comparative effectiveness, is recommended in order to avoid duplication and contributing to more equitable access to medicines (23–26). This is particularly crucial given the continuous strain on healthcare budgets, even in strong economies, such as the EU, and the significant affordability challenges affecting patients’ access to necessary treatments. Differences in the availability of health technologies for patients across EU countries are evident (27), emphasizing the urgency of addressing these disparities.

As the journey continues, the EUnetHTA 21 experience underscores a shared vision of HTA bodies aligning with the HTAR. This vision aims to enhance the quality of HTA, avoid duplication, ensure the lasting sustainability of EU-HTA cooperation, and ultimately contribute to improving patient access to the safest, most effective and affordable health technologies for all EU patients in a timely and equitable manner.
